# Analysis of credit linked demand in an inventory model with varying ordering cost

**DOI:** 10.1186/s40064-016-2567-9

**Published:** 2016-06-29

**Authors:** Ateka Banu, Shyamal Kumar Mondal

**Affiliations:** Department of Applied Mathematics with Oceanology and Computer Programming, Vidyasagar University, Midnapore, W.B. 721 102 India

**Keywords:** Inventory, Deterioration, Two-level credit financing, Credit period dependent demand, Inflation

## Abstract

In this paper, we have considered an economic order quantity model for deteriorating items with two-level trade credit policy in which a delay in payment is offered by a supplier to a retailer and also an another delay in payment is offered by the retailer to his/her all customers. Here, it is proposed that the demand function is dependent on the length of the customer’s credit period and also the duration of offering the credit period. In this article, it is considered that the retailer’s ordering cost per order depends on the number of replenishment cycles. The objective of this model is to establish a deterministic EOQ model of deteriorating items for the retailer to decide the position of customers credit period and the number of replenishment cycles in finite time horizon such that the retailer gets the maximum profit. Also, the model is explained with the help of some numerical examples.

## Background

In traditional business system, it is seen that customers must pay as soon as they receive the purchase quantity. But, recently, seller may offer a delay period to pay their dues which is known as credit period. Actually, this credit period is the time frame between when a customer purchases a product and when the customer’s payment is made. The credit policy helps to build up a long term relationship of a company with his customers and it also increases the customers demand. Now-a-days, the trade credit financing in the business becomes a trend due to its various advantages. The effect of credit period in traditional EOQ system first was studied by Goyal (1985). Chung (1998) simplified the search of the optimal solution for the problem explored by Goyal (1985). After that so many researchers also worked on constant or variable demand with credit period such as Banerjee (1986), Ha and Kim (1997), Ouyang et al. (2004), Das et al. (2007). Huang (2003) developed an EOQ model in which a supplier offers a retailer the permissible delay period *M* and the retailer also provides the trade credit period *N* ($N\le M$) to his/her customers. Some EOQ model have been developed for items under permissible delay in payment depending on ordering quantity. Shinn and Hwang (2003) extended the work of Shinn (1997) in optimal pricing and ordering policies for retailers under order-size dependent delay in payment. Teng et al. (2011) extended an EOQ model for stock-dependent demand to supplier’s trade credit with a progressive payment scheme. Lou and Wang (2013) developed the classical EOQ model considering credit-linked demand and default risks. Later, Das et al. (2014) discussed a transportation problem in an integrated production-inventory model with a discrete credit period. More recent articles on trade credit financing are developed by Chen and Kang (2010), Chung and Cardenas-Barron (2013), Yang et al. (2014), Chern et al. (2013), Das et al. (2013), Cardenas-Barron et al. (2014), Wu and Chan (2014), Chung et al. (2014), Dye and Yang (2015), Benkherouf and Gilding (2015), Shah and Cardenas-Barron (2015) etc. Recently, Das et al. (2015) developed an integrated model with fuzzy credit period and deterioration.

Practically, it is seen that the longer credit period attracts more customers. So, *to increase the length of credit period increases the customers’ demand*. From the literature survey on credit periods, it is studied that almost all research works have considered one manufacturer/supplier to offer the credit period to only one retailer. And there are also very few papers (Dye and Ouyang 2011) in which the retailer offers the credit period to end customer (consumer). In their paper, only one end customer has been considered. But, it is impractical to consider only one customer by which all items are consumed together. It is happened that there are many end customers by which items are procured from the retailer at different times during the business period and it is also observed that sometimes the retailer announces a credit period during some period to fascinate more end customers.* But, till now no one has considered demand as a function of customer’s credit and duration of offering the credit period.* In this paper, this phenomena has been incorporated.

In real life situation, the deterioration of items is a natural phenomena which is normally caused by vaporization, damage, spoilage, dryness, poor preservation technology etc, take place frequently in inventory system and cause great losses to inventory managers. During the past few decades, a lot of model with deterioration have published. Ghare and Schrader (1963) were the first proponents to establish a model for an exponentially decaying inventory. Aggarwal and Jaggi (1995) and Hwang and Shinn (1997) extended the model of Goyal considering the deterministic inventory model constant deterioration rate. Jamal et al. (1997) further generalized the model to allow the shortages. Taso and Sheen (2007) developed a finite time horizon model for deteriorating items to determine the most suitable retail price price and appropriate replenishment cycle time with fluctuating demand. Subsequently, several researchers develop inventory model concerning deterioration such as Wu et al. (2014), Sarkar et al. (2015) and Wu et al. (2016) etc.

Many research papers are developed without considering inflation rate as most decision makers think that the inflation have no significant effect on inventory policy. But, from financial point of view, an inventory represents a capital investment and must compete with other assets for a firm’s limited capital fund. Therefore, it is important to investigate how inflation and time value of money affect on various inventory policies. Trippi and Lewin (1974) discussed a cash discount flow approach to obtain the present value of average inventory costs in an infinite time horizon. Dohi et al. (1992) developed a inventory model with and without backlogging allowed for an infinite time horizon considering time value of money. Bose et al. (1995) discussed an inventory model with time value of money and inflation for deteriorating items. There are so many papers with inflation and time value of money under different field such as Moon and Yun (1993), Hariga (1994), Chung and Lin (2001), Dye and Ouyang (2011) etc. Liao et al. (2000) developed a model for deteriorating items considering inflation when a delay payment is permissible. Recently, Gilding (2014) developed a inventory model in a finite time horizon considering inflation.

From the literature review, regarding the inventory models in fixed time horizon, it is seen that the ordering cost is always fixed for all cycles. But, realistically it is not correct. Basically, when a retailer orders the materials to the supplier, then there exists two types of cost such as fixed cost and variable cost. The fixed cost consists of the cost related to facilities, telephone and maintenance of computer system to process the purchase orders. The variable cost is the cost related to the shipments of the purchase quantities. Obviously this type of cost depends on number of replenishment cycles to be processed as when the number of replenishment cycle is increased the ordering amount decreases per cycle or vice-versa.

From the observations in the existing literature in which the ordering cost is fixed per cycle, it is seen that whenever the number of replenishment cycles is more, the ordering cost be very high than the ordering cost in one cycle though in each case total quantity delivered is same in finite time horizon. Practically, in real business world, this concept is not completely error free. Again, whenever number of replenishment cycles is more then the shipment cost is reduced inversely since the quantity delivered is less per cycle. That is, if $a_1$ be the shipment cost for total quantity *Q* to be delivered in one time in the business period then for *n* replenishment, the shipment cost per cycle will be $a_1/n$, since in this case the total quantity *Q* is delivered in *n* cycle of amount *Q*/*n* per cycle. So, the shipment cost per cycle varies inversely with *n*. Again, if the total quantity *Q* is delivered in more than one cycle, clearly due to the processing the delivery supplier must claim an extra charge which is known as processing cost. Notedly, this type of cost will be increased whenever number of replenishment cycles also increases. That is, the ordering cost can not be constant for a fixed time horizon.* But, till now, no one has considered variable ordering cost depending on replenishment cycle in a fixed time horizon*.

In this paper, an EOQ model for deteriorating items with inflation in a finite time horizon has been developed with two level credit financing. One credit is offered by supplier to retailer and another credit is offered by retailer to his/her all customers. Here, we have considered demand as a function of length of the customer’s credit as well as its duration of offering and time in exponential form and ordering cost as replenishment cycle number dependent function. Also, we have assumed that all customers take the advantages of same credit period upto a limited period. We then characterize the customer’s optimal credit period and cycle number.

## Notations and assumptions

To formulate this model we have used the following notations and assumptions.

### Notations

(i) *I*(*t*): the inventory level at time *t*.
(ii) *D*(*t*): the demand function at time *t*.
(iii) *H*: total planning horizon (in year).
(iv) *A*: retailer’s ordering cost per order.
(v) *p*: retailer’s purchase cost per unit item.
(vi) *s*: retailer’s selling price per unit item .
(vii) *r*: rate of inflation.
(viii) $\theta$: rate of deterioration.
(ix) *h*: retailer’s holding cost per unit per unit time.
(x) $I_e$: rate of interest earned.
(xi) $I_c$: rate of interest charged for delay payment after offered credit period.
(xii) *M*: the retailer’s trade credit period offered by the supplier in years.
(xiii) *N*: the customers credit period offered by the retailer in years, where $N\le M$ (a decision variable).
(xiv) *n*: the number of replenishment cycles during the planning horizon (a decision variable).
(xv) $[t_{i-1},t_i]$: the *i*th replenishment cycle, $i=1,2,3,\ldots,n$.
(xvi) $Q_i$: the order quantity in the *i*th replenishment period.
(xvii) *TPj*: total profit of the retailer, $j=1,2,3$.

### Assumptions

(i) The inventory system involves only one item over a finite planning horizon *H*.
(ii) The replenishment occurs instantaneously at an infinite rate.
(iii) Shortages are not allowed.
(iv) The items deteriorate at a constant rate of deterioration $\theta$, where $0<\theta \le 1$. There is no repair or replacement of deteriorated units during the planning horizon.
(v) Here, two credit periods have been considered in each cycle. One (*M*) of them is offered by a supplier to the retailer and another one (*N*) is offered by the retailer to each customer in such a way that each customer must pay within the period of the credit period (*M*) offered by the supplier to the retailer. It is also assumed that the customer’s credit period is less than or equal to the retailer’s credit period i.e., $N\le M$.
(vi) It is considered that the retailer pay his/her dues at the end of each cycle. So, if the length of the replenishment cycle is greater than the length of the credit period the retailer has to an interest at a rate $I_c$ . If the length of replenishment cycle is less than the length of the credit period then no interest is charged.
(vii) In this model, a retailer intends to offer a credit period (*N*) (decision variable) to each customer in certain duration to increase his/her demand (*D*(*t*)). Here, the duration of credit period is proposed in such a way that the last end customer who takes the facility of credit period pay his/her dues at the time of credit period (*M*) offered by the supplier . Therefore, all end customers having this facility, come during the period $(0,M-N)$ in each cycle. As $M-N$ is large, so number of customers takes this privilege. That is, the demand depends on both *N* and $M-N$. For these reasons, the demand function *D*(*t*) has been considered as a exponential function of time in respect of $N(M-N)$ which is defined as follows:
  $$\begin{aligned} D(t)&=\left\{ \begin{array}{ll} D_0 e^{b_1N(M-N)t}; &{}\quad \text{ when }\; t_{i-1}\le t\le t_{i-1}+(M-N) \\ D_0e^{b_2N(M-N)(t_{i-1}+M-N)}; &{}\quad \text{ when }\; t_{i-1}+(M-N) \le t\le t_i \end{array}\right. \end{aligned}$$
  where $D_0>0$ is a scaling parameter and $b_1$, $b_2$ ($b_1\ge b_2$) are positive, which are known as effective parameters for credit periods.
(viii) All replenishment cycles have same size.
(ix) In this paper, it is assumed that the ordering cost (*A*) per cycle has been proposed in the following way:
  $$A = a_0+\frac{a_1}{n}+a_2(n-1)$$
  where, *a*_0_ (>0) be the fixed cost per cycle, *a*_1_ (>0) be the shipment cost for the total quantity delivered in one time and *a*_2_ (>0) be the process cost per cycle. Also, here it is clear that for *n* cycles the total ordering cost considering fixed ordering cost per cycle must be greater than the variable ordering cost per cycle depending on the processing cost. That is, considering fixed ordering cost per cycle total ordering cost (*TFOC*) will be greater than the total ordering cost (*TVOC*) considering variability in the ordering cost provided that
  $$\begin{aligned}&\quad n(a_0+a_1)>n\left[ a_0+\frac{a_1}{n}+a_2(n-1)\right] \\ \text{ or, }&\quad a_1>a_0+\frac{a_1}{n}+a_2(n-1) \\ \text{ or, }&\quad(n-1)\left( \frac{a_1}{n}\right)>0 \\ \text{ or, }&\quad\frac{a_1}{n}>a_2 \end{aligned}$$
  From this following lemma can be drawn:

#### **Lemma 1**

*For n cycles, TFOC must be greater thanTVOC provided that *$\frac{a_1}{n}>a_2$.

## Mathematical model formulation

**Fig. 1:**
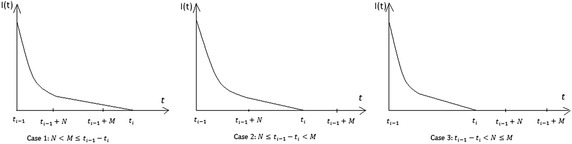
Retailer’s inventory level

In this model, the retailer first receives $Q_i$ amount items from supplier and then fulfils the demands of his/her customers from his stock during the time period $[t_{i-1},t_i]$ and this process continues up-to end of the fixed time horizon *H*. Here, the supplier offers credit period to the retailer and the retailer also offers credit periods to each customer during the time period $[t_{i-1},t_{i-1}+M-N]$ according to assumption (v). After that no credit will be given to the customers. So, in this problem three possibilities may arise due to different positions of M and N with the interval such as: Case 1: $N<M\le t_i-t_{i-1}$, Case 2: $N\le t_i-t_{i-1}<M$ and Case 3: $t_i-t_{i-1}<N\le M$. The retailer's inventory level for the different three cases are shown in Fig. 1.

### Model formulation of the retailer

In each cycle,deterioration and demand both effect on the inventory. Hence the differential equation of the inventory level *I*(*t*) in the *i*th cycle for the retailer is given by

$$\begin{aligned} \frac{dI(t)}{dt}&=\left\{ \begin{array}{llll} -\theta {I(t)}-D_0 e^{b_1N(M-N)t};&{}\quad \text{ when }\; t_{i-1}\le t < t_{i-1}+(M-N) \\ -\theta {I(t)}-D_0e^{b_2N(M-N)(t_{i-1}+M-N)};&{}\quad \text{ when }\; t_{i-1}+(M-N)\le t\le t_i \end{array}\right. \end{aligned}$$

with the boundary conditions

1 $$I(t_{i-1})=Q_i ,\; I(t_i)=0; \quad i=1,2,\ldots ,n,$$

Solving the differential Eq. (1) we get,

$$\begin{aligned} I(t) &=\left\{ \begin{array}{ll} Q_ie^{\theta (t_{i-1}-t)}-\frac{D_0e^{-\theta {t}}}{b_1N(M-N)+\theta } \left\{ e^{(b_1N(M-N)+\theta )t}-e^{(b_1N(M-N)+\theta )t_{i-1}}\right\} ;&{}\text{ when }\; t_{i-1}\le t < t_{i-1}+(M-N) \\ \frac{D_0}{\theta }e^{b_2N(M-N)(t_{i-1}+M-N)} \left\{ e^{\theta {(t_i-t)}}-1\right\} ; &{}\text{ when }\; t_{i-1}+(M-N) \le t \le t_i \\ \end{array}\right. \end{aligned}$$

where

$$\begin{aligned} Q_i &=\frac{D_0}{b_1N(M-N)+\theta }\left[ e^{(b_1N(M-N)+\theta )(M-N)}-1\right] e^{b_1N(M-N)t_{i-1}} \\ &\quad+\frac{D_0}{\theta }\left[ e^{\theta (t_i-(t_{i-1}+M-N))}-1\right] e^{b_2N(M-N)(t_{i-1}+M-N)+\theta (M-N)} \end{aligned}$$

#### *Holding cost *

The present value of the holding cost $(H_{i})$, $i=1,2,3,\ldots ,n$, in the *i*th cycle is given by

2 $$\begin{aligned} H_i & = h\left[ \int _{t_{i-1}}^{t_{i-1}+M-N}e^{-rt}I(t)dt+\int _{t_{i-1}+M-N}^{t_i}e^{-rt}I(t)dt\right] \\ &= h\left[ \frac{Q_ie^{\theta t_{i-1}}}{r+\theta }\left\{ e^{-(r+\theta )t_{i-1}}-e^{-(r+\theta )(t_{i-1}+M-N)}\right\} \right. \\&\quad -\,\frac{D_0}{(b_1N(M-N)+\theta )(b_1N(M-N)-r)}\left\{ e^{(b_1N(M-N)-r)(t_{i-1}+M-N)}-e^{(b_1N(M-N)-r)t_{i-1}}\right\} \\&\quad \left. -\,\frac{D_0e^{(b_1N(M-N)+\theta )t_{i-1}}}{(b_1N(M-N)+\theta )(r+\theta )}\left\{ e^{-(r+\theta )(t_{i-1}+M-N)}-e^{-(r+\theta )t_{i-1}}\right\} \right] \\&\quad +\,\frac{hD_0}{\theta }e^{b_2N(M-N)(t_{i-1}+M-N)}\left[ \frac{1}{r}\left\{ e^{-rt_i}-e^{-r(t_{i-1}+M-N)}\right\} \right. \\&\quad \left. -\,\frac{e^{\theta t_i}}{r+\theta }\left\{ e^{-(r+\theta )t_i}-e^{-(r+\theta )(t_{i-1}+M-N)}\right\} \right] \ \end{aligned}$$

#### *Purchase cost*

The present value of the purchase cost during the *i*th replenishment period, denoted by $P_i$, $i=1,2,3,\ldots ,n$, is

3 $$\begin{aligned} P_i & = pe^{-rt_{i-1}}I(t_{i-1}) \\ &= \frac{pD_0}{b_1N(M-N)+\theta }\left[ e^{(b_1N(M-N)+\theta )(M-N)}-1\right] e^{(b_1N(M-N)-r)t_{i-1}} \\&\quad +\,\frac{pD_0}{\theta }\left[ e^{\theta (t_i-(t_{i-1}+M-N))}\right] e^{b_2N(t_{i-1}+M-N)+\theta (M-N)-rt_{i-1}} \end{aligned}$$

#### *Sales revenue*

The present value of the sales revenue in the *i*th replenishment period, denoted by $S_i$, $i=1,2,3,\ldots ,n$, is given by

4 $$\begin{aligned} S_i &= s\left[ \int _{t_{i-1}}^{t_{i-1}+M-N}e^{-rt}D(t)dt+\int _{t_{i-1}+M-N}^{t_i}e^{-rt}D(t)dt\right] \\ &= \frac{sD_0}{b_1N(M-N)-r}\left[ e^{(b_1N(M-N)-r)(t_{i-1}+M-N)}-e^{(b_1N(M-N)-r)t_{i-1}}\right] \\&\quad -\,\frac{sD_0}{r}e^{b_2N(M-N)(t_{i-1}+M-N)}\left[ e^{-rt_i}-e^{-r(t_{i-1}+M-N)}\right] \end{aligned}$$

#### **Case 1**

 When $$N<M\le t_i-t_{i-1}$$

In this case, it is assumed that length of the replenishment period is greater than the retailer’s credit period, i.e., here, $N<M\le H/n$. Also in this case, it is considered that retailer will pay his dues at the end of the replenishment cycle and therefore he has to pay an interest at a rate $I_c$ to the supplier.

#### *Interest earned*

The present value of the interest earned in the *i*th replenishment period, denoted by ${\textit{ IE}}_{i1}$, $i=1,2,3,\ldots ,n$, is given by

5 $$\begin{aligned} {\textit{IE}}_{i1} &= I_es\left[ \int _{t_{i-1}}^{t_{i-1}+M-N}e^{-rt}D(t)[t_i-(t+N)]dt+\int _{t_{i-1}+M-N}^{t_i}e^{-rt}D(t)[t_i-t]dt\right] \\&= \frac{I_esD_0(t_i-N)}{b_1N(M-N)-r}\left[ e^{(b_1N(M-N)-r)(t_{i-1}+M-N)}-e^{(b_1N(M-N)-r)t_{i-1}}\right] \\&\quad -\,\frac{I_esD_0}{b_1N(M-N)-r}\left[ \left\{ (t_{i-1}+M-N)e^{(b_1N(M-N)-r)(t_{i-1}+M-N)}-t_{i-1}e^{(b_1N(M-N)-r)t_{i-1}}\right\} \right. \\&\quad \left. -\,\frac{1}{b_1N(M-N)-r}\left\{ e^{(b_1N(M-N)-r)(t_{i-1}+M-N)}-e^{(b_1N(M-N)-r)t_{i-1}}\right\} \right] \\&\quad -\,\frac{sI_eD_0t_i}{r}e^{b_2N(M-N)(t_{i-1}+M-N)}\left[ e^{-rt_i}-e^{-r(t_{i-1}+M-N)}\right] \\&\quad +\,\frac{sI_eD_0}{r}e^{b_2N(M-N)(t_{i-1}+M-N)}\left[ \left\{ t_ie^{-rt_i}-(t_{i-1}+M-N)e^{-r(t_{i-1}+M-N)}\right\} \right. \\&\quad \left. +\,\frac{1}{r}\left\{ e^{-rt_i}-e^{-r(t_{i-1}+M-N)}\right\} \right] \end{aligned}$$

#### *Interest charged*

 The present value of interest charge in the *i*th replenishment period, denoted by ${\textit{IC}}_{i1}$, $i=1,2,3,\ldots ,n$, is given by

6 $$\begin{aligned} {\textit{IC}}_{i1} &= pI_c\left[ \int _{t_{i-1}+M}^{t_i}e^{-rt}I(t)dt\right] \\ &= pI_c\left[ \int _{t_{i-1}+M}^{t_i}e^{-rt}\frac{D_0}{\theta }e^{bN(M-N)(t_{i-1}+M-N)}\left[ 1-e^{\theta (t_i-t)}\right] dt\right] \\ &= \frac{pI_cD_0}{\theta }e^{b_2N(M-N)(t_{i-1}+M-N)}\left[ \frac{1}{r}\left\{ e^{-rt_i}-e^{-r(t_{i-1}+M)}\right\} \right. \\&\quad\left. -\frac{e^{\theta t_i}}{r+\theta }\left\{ e^{-(r+\theta )t_i}-e^{-(r+\theta )(t_{i-1}+M)}\right\} \right] \end{aligned}$$

#### *Total profit*

So, in this case, retailer’s total profit ${\textit{TP1}}(n,N)$ can be expressed as

$$\begin{aligned} {\textit{TP1}}(n,N) &= \text{ sales } \text{ revenue } - \text{ purchase } \text{ cost } - \text{ holding } \text{ cost } - \text{ interest } \text{ charge } \\&\quad +\text{ interest } \text{ earned } - \text{ ordering } \text{ cost } \end{aligned}$$

i.e.,

7 $$\begin{aligned} \textit{TP1}(n,N) &= \sum _{i=1}^{n}\left( S_i-P_i-H_i-IC_{i1}+IE_{i1}-e^{-rt_{i-1}}A\right) \\ &= \left[ \left\{ \frac{sD_0}{b_1N(M-N)-r}-\frac{hD_0}{(b_1N(M-N)-r)(r+\theta )}\right\} \left[ e^{(b_1N(M-N)-r)(M-N)}-1\right] \right. \\&\quad -\left\{ \frac{pD_0}{b_1N(M-N)+\theta }+\frac{hD_0[1-e^{-(r+\theta )(M-N)}]}{(r+\theta )(b_1N(M-N)+\theta )}\right\} \left[ e^{(b_1N(M-N)+\theta )(M-N)}-1\right] \\&\quad +\frac{hD_0}{r+\theta }\left\{ \frac{1}{b_1N(M-N)+\theta }+\frac{1}{\theta }\left[ e^{\theta (H/n-M+N)}-1\right] e^{(b_1N(M-N)+\theta )(M-N)}\right\} \\&\qquad \left. \left[ e^{-(r+\theta )(M-N)}-1\right] \right] \frac{1-e^{(b_1N(M-N)-r)H}}{1-e^{(b_1N(M-N)-r)H/n}} \\&\quad +\left[ \left( \frac{sD_0}{r}+\frac{hD_0}{r\theta }\right) \left\{ e^{(b_2N(M-N)-r)(M-N)}-e^{b_2N(M-N)^2-rH/n}\right\} \right. \\&\quad -\,\frac{pD_0}{\theta }\left\{ e^{\theta (H/n-M+N)-1}\right\} e^{(b_2N(M-N)+\theta )(M-N)}+\frac{hD_0}{\theta (r+\theta )}\left\{ e^{b_2N(M-N)^2-rH/n}\right. \\&\quad \left. \left. -\,e^{(b_2N(M-N)-r-\theta )(M-N)+\theta H/n}\right\} \right] \frac{1-e^{(b_2N(M-N)-r)H}}{1-e^{(b_2N(M-N)-r)H/n}} \\&\quad +\frac{sI_eD_0}{{b_1N(M-N)-r}}\left[ (H/n-M)e^{(b_1N(M-N)-r)(M-N)}-(H/n-N)\right. \\&\quad \left. +\,\frac{1}{(b_1N(M-N)-r)}\left[ e^{(b_1N(M-N)-r)(M-N)}-1\right] \right] \frac{1-e^{(b_1N(M-N)-r)H}}{1-e^{(b_1N(M-N)-r)H/n}} \\&\quad +\frac{sI_eD_0}{r}\left[ (H/n-M+N)e^{(b_2N(M-N)-r)(M-N)}+\frac{1}{r}\left[ e^{b_2N(M-N)^2-rH/n}\right. \right. \\&\quad \left. \left. -\,\,e^{(b_2N(M-N)-r)(M-N)}\right] \right] \frac{1-e^{(b_2N(M-N)-r)H}}{1-e^{(b_2N(M-N)-r)H/n}}-\frac{pI_cD_0}{\theta } \left[ \frac{1}{r+\theta }\left[ e^{b_2N(M-N)^2-(r+\theta )M+\theta H/n}\right. \right. \\&\quad \left. \left. -\,e^{b_2N(M-N)^2-rH/n}\right] +\frac{1}{r}\left[ e^{b_2N(M-N)^2-rH/n}-e^{b_2N(M-N)^2-rM}\right] \right] \frac{1-e^{(b_2N(M-N)-r)H}}{1-e^{(b_2N(M-N)-r)H/n}} \\&\quad -\,A\frac{1-e^{-rH}}{1-e^{-rH/n}}\ \end{aligned}$$

#### **Case 2**

When $$N\le t_i-t_{i-1}<M$$

In this case, it is assumed that the length of the replenishment period is less than the retailer’s credit period. So no interest is charged from the retailer i.e. ${\textit{IC}}_{i2}=0$. Also the retailer earns interest from the customers during the period $[t_{i-1}+N,t_{i-1}+M]$.

#### **Lemma 2**

$$M-N\le H/n<M$$.

#### Proof

Since the retailer offers credit period *N* to each customer in such a way that all customers must pay their dues within the retailer’s credit period, so in this case, the following must be hold for *i*th cycle.

$$\begin{aligned}&\quad t_{i-1}+M-N\le t_i \\ \text{ i.e., }&\quad M-N\le t_i-t_{i-1} \\ \text{ i.e., }&\quad M-N\le t_i-t_{i-1}<M \\ \text{ i.e., }&\quad M-N\le H/n<M \end{aligned}$$

#### *Interest earned*

 Thus the present value of interest earned,denoted by $\textit{IE}_{i2}$, $i=1,2,3,\ldots ,n$, is given by

8 $$\begin{aligned} \textit{IE}_{i2}&= I_es\left[ \int _{t_{i-1}}^{t_{i-1}+M-N}e^{-rt}D(t)[(t_{i-1}+M)-(t+N)]dt+\int _{t_{i-1}+M-N}^{t_i}e^{-rt}D(t)(t_{i-1}+M-t)dt\right] \\ &= \frac{sI_eD_0}{b_1N(M-N)-r}(N-M)e^{(b_1N(M-N)-r)t_{i-1}} \\&\quad +\,\frac{sI_eD_0}{(b_1N(M-N)-r)^2}\left[ e^{(b_1N(M-N)-r)(t_{i-1}+M-N)}-e^{(b_1N(M-N)-r)t_{i-1}}\right] \\&\quad +\,\frac{sI_eD_0}{r}\left[ (H/n-M)e^{b_2N(M-N)(t_{i-1}+M-N)-rt_i}+Ne^{(b_2N(M-N)-r)(t_{i-1}+M-N)}\right] \\&\quad +\,\frac{sI_eD_0}{r^2}e^{b_2N(M-N)(t_{i-1}+M-N)}\left[ e^{-rt_i}-e^{-r(t_{i-1}+M-N)}\right] \end{aligned}$$

#### *Total profit*

 So, in this case, the retailer’s total profit $\textit{TP}2(n,N)$ can be expressed as

$$\begin{aligned} \textit{TP2}(n,N) &= \text{ sales } \text{ revenue } - \text{ purchase } \text{ cost } - \text{ holding } \text{ cost } - \text{ interest } \text{ charge } \\&\quad +\, \text{ interest } \text{ earned } - \text{ ordering } \text{ cost } \end{aligned}$$

i.e.,

9 $$\begin{aligned} \textit{TP2}(n,N) &= \sum _{i=1}^{n}(S_i-P_i-H_i+IE_{i2}-e^{-rt_{i-1}}A) \\ &= \left[ \left\{ \frac{sD_0}{b_1N(M-N)-r}-\frac{hD_0}{(b_1N(M-N)-r)(r+\theta )}\right\} \left[ e^{(b_1N(M-N)-r)(M-N)}-1\right] \right. \\&\quad -\,\left\{ \frac{pD_0}{b_1N(M-N)+\theta }+\frac{hD_0[1-e^{-(r+\theta )(M-N)}]}{(r+\theta )(b_1N(M-N)+\theta )}\right\} \left[ e^{(b_1N(M-N)+\theta )(M-N)}-1\right] \\&\quad +\,\frac{hD_0}{r+\theta }\left\{ \frac{1}{b_1N(M-N)+\theta }+\frac{1}{\theta } \left[ e^{\theta (H/n-M+N)}-1\right] e^{(b_1N(M-N)+\theta )(M-N)}\right\} \\&\qquad \left. \left[ e^{-(r+\theta )(M-N)}-1\right] \right] \frac{1-e^{(b_1N(M-N)-r)H}}{1-e^{(b_1N(M-N)-r)H/n}} \\&\quad +\left[ \left( \frac{sD_0}{r}+\frac{hD_0}{r\theta }\right) \left\{ e^{(b_2N(M-N)-r)(M-N)}-e^{b_2N(M-N)^2-rH/n}\right\} \right. \\&\quad -\frac{pD_0}{\theta }\left\{ e^{\theta (H/n-M+N)-1}\right\} e^{(b_2N(M-N)+\theta )(M-N)}+\frac{hD_0}{\theta (r+\theta )}\left\{ e^{b_2N(M-N)^2-rH/n}\right. \\&\quad \left. \left. -e^{(b_2N(M-N)-r-\theta )(M-N)+\theta H/n}\right\} \right] \frac{1-e^{(b_2N(M-N)-r)H}}{1-e^{(b_2N(M-N)-r)H/n}} \\&\quad +\frac{sI_eD_0}{b_1N(M-N)-r}\left[ (N-M)+\frac{1}{b_1N(M-N)-r}\left[ e^{(b_1N(M-N)-r)(M-N)}\right. \right. \\&\quad \left. \left. -1\right] \right] \frac{1-e^{(b_1N(M-N)-r)H}}{1-e^{(b_1N(M-N)-r)H/n}}+\frac{sI_eD_0}{r}\left[ \left\{ (H/n-M)e^{b_2N(M-N)^2-rH/n}+Ne^{(b_2N(M-N)-r)(M-N)}\right\} \right. \\&\quad \left. +\frac{1}{r}\left\{ e^{b_2N(M-N)^2-rH/n}-e^{(b_2N(M-N)-r)(M-N)}\right\} \right] \frac{1-e^{(b_2N(M-N)-r)H}}{1-e^{(b_2N(M-N)-r)H/n}}-A\frac{1-e^{-rH}}{1-e^{-rH/n}}\ \end{aligned}$$

#### **Case 3**

 When $$t_i-t_{i-1}<N\le M$$

In this case, it is assumed that the length of the replenishment period is less than the retailer’s credit period. So no interest is charged from retailer i.e. ${\textit{IC}}_{i3}=0$ and earns interest from the customer during the period $[t_{i-1}+N,t_{i-1}+M]$.

#### **Lemma 3**

$$M-N\le H/n<N$$.

#### Proof

Since all customers must pay their dues within the retailer’s credit period, so the following must be hold for *i*th cycle.

$$\begin{aligned}&\quad t_{i-1}+M-N\le t_i \\ \text{ i.e., }&\quad M-N\le t_i-t_{i-1}< N \\ \text{ i.e., }&\quad M-N\le H/n<N \end{aligned}$$

In this case, the expression of the total profit $\textit{TP3}(n,N)$ is the same as the expression of the total profit in Case 2 considering only $\textit{IC}_{i3}=0$.

## Numerical illustrations

To illustrate the above model, the following numerical examples have been considered.

### *Problem-1* (*Variable ordering cost*)

A company supplies one kind of items to a retailer at cost of $35 per unit item and offers a credit period 50/365 to the retailer in such a way that he will enjoy the relaxation on interest charge during this period. In this business policy it has been settled that the retailer pays his dues at the end of each cycle. If the credit period be less than the cycle length then due to late payment the retailer must pay an interest to the supplier at a rate of 8 %. The retailer’s holding cost is $3 per unit item and the items deteriorate at a rate of 1 %. The retailer sales each unit of items at cost of $50 to customers. The retailer also offers same credit *N* to each customer upto a certain time in such a way that all customers must pay their dues within the retailer’s credit period, after that no credit will be given and the retailer earns interest at a rate of 6 %. The ordering cost of the retailer has been considered according to assumption (ix). Here, the retailer’s objective is to maximize the total profit. Find the optimal number replenishment cycle period and optimal credit period *N* offered by the retailer.

### *Solution*

 In this problem, $$a_0=200$$, $$a_1=1000$$; $$a_2=30$$, $$b_1=10$$, $$b_2=5$$$$D_0=1000$$, $$h=3$$, $$r=0.1$$, $$\theta =0.01$$, $$H=1$$, $$p=35$$, $$s=50$$, $$M=50/365$$, $$I_c=0.08$$, $$I_e=0.06$$.

**Fig. 2:**
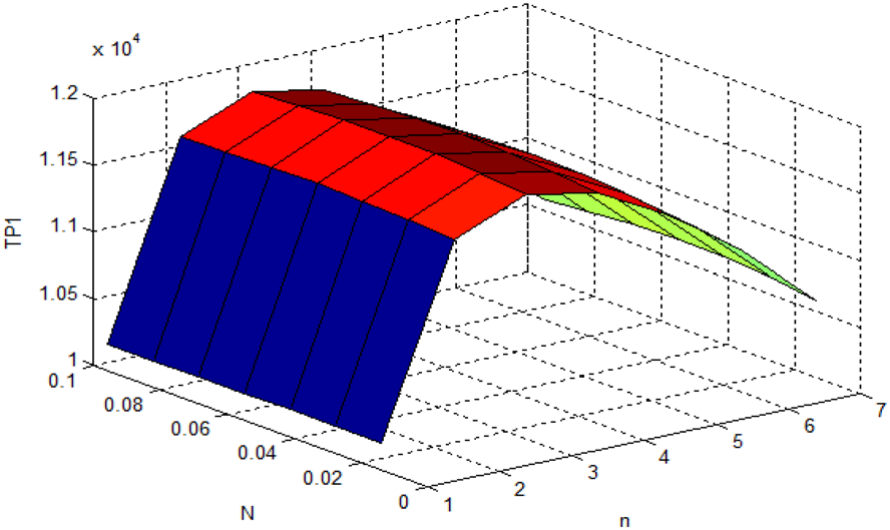
Concavity of *TP*1

Since the model corresponding to this problem is non-linear, it cannot be optimized analytically. So to get the optimal solution, the standard LINGO software has been used of such type of model utilizing the above parameters. Before getting the optimal solution, the concavity of the objective function has been shown by Fig. 2. The obtained results have been shown in the Table 1.

**Table 1:** The results of Problem-1 and Problem-2

	Variable ordering cost			Fixed ordering cost		
	Case 1	Case 2	Case 3	Case 1	Case 2	Case 3
*N*	0.0558	0.0494	0.1250	0.0559	0.0494	0.1250
*n*	3	8	8	2	8	8
Profit (*TPj*)	11896.19	10132.84	9997.08	10739.05	5038.48	4902.73

### *Problem-2* (*Fixed ordering cost*)

 The same problem in Problem-1 when retailer consider ordering cost as a fixed value, $ 1200 which is same as the ordering cost when replenishment cycle number is 1 for the variable ordering cost.

### *Solution*

 The obtained results for three cases have been shown in Table 1.

### *Problem-3* (*For constant demand*)

 The same problem in Problem-1 when constant demand is considered i.e., when $b_1=0=b_2$ for both the cases considering fixed ordering cost and variable ordering cost.

### Solution

The obtained results have been shown in Table 2.

**Fig. 3:**
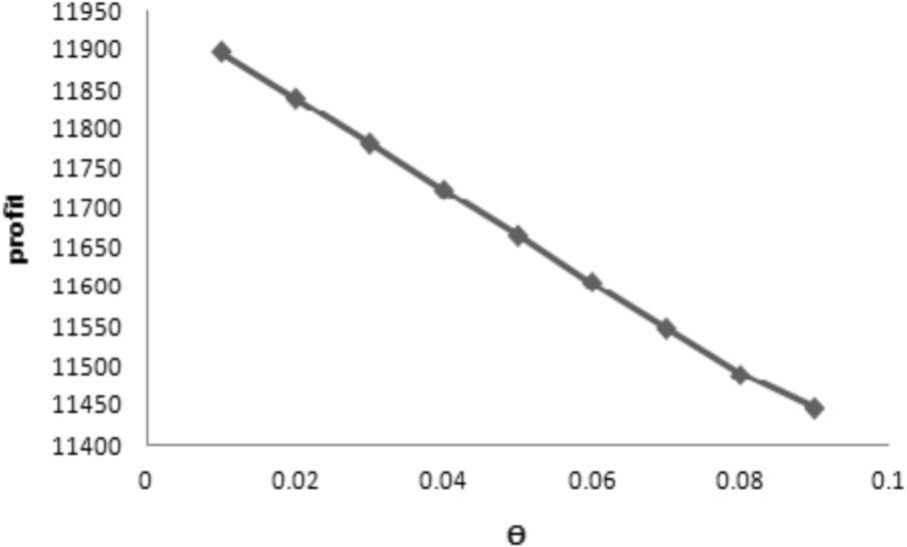
*θ* versus profit for Case 1

From results obtained in Tables 1, 2, 3, 4, 5 and from Fig. 3 following managerial insights have been drawn:

**Table 2:** The results of Problem-3

	Variable ordering cost			Fixed ordering cost		
	*N*	*n*	Profit (*TPj*)	*N*	*n*	Profit (*TPj*)
Case 1	0.1369	3	11783.21	0	2	10655.33
Case 2	0.125	8	9946.09	0.125	8	4851.74
Case 3	0.1369	8	9980.49	0.1369	8	4886.15

- From Table 1, it is observed that when variable ordering cost is considered then Case 1 is most profitable other than Case 2 and Case 3. In this case, optimal profit and the optimal values of the variables are $TP1=11896.19$, $N=0.0558$ and $n=3$. Again, when fixed ordering cost is considered from Table 1, it is seen that Case 1 is also profitable other than the two cases . So, investigating Table 1, it is concluded that model with variable ordering cost is profitable than the model with fixed ordering cost.
- Considering constant demand i.e., when $b_1=0=b_1$ from Table 2, it is seen that for the both cases Case 1 is most profitable with profit $TP1=11783.21$, $n=3$ for variable ordering cost and $TP1= 10655.33$, $n=2$ for fixed ordering cost. But, offering credit period by the retailer is large ($N=0.1369$) for the case considering variable ordering cost whereas for the case considering fixed ordering cost it is zero. That is the case considering fixed ordering cost for constant demand is profitable when no credit will be given by the retailer.
- From Table 3, it is investigated that with the increase of inflation rate the optimal profit decreases with customer’s credit period, but number of replenishment cycles increases.
- Figure 3 shows the change of profit with respect to *θ*. From this, it is observed that the optimal total profit decreases with the increase of *θ* which is normal.
- From Table 4, it is seen that when the credit period (*M*) offered by the supplier to the retailer increases, then the optimum profit of the model also increases along with increasing the customer’s credit period (*N*) though the number of replenishment cycle is almost same. Thus, the retailer may offer more credit to his/her customer to get more profit.
- In Table 5, it is observed that on increasing processing cost $a_2$ profit *TP*1 decreases but offering credit period *N* remains same and the number of replenishment cycles also decreases. But, it is more profitable than the case when fixed ordering cost is considered according to Lemma 1.

**Table 3:** Variation of results for different *r* for Case 1

*r*	*N*	*n*	TP1	*Q*
0.1	0.0558	3	11,896.19	1013.02
0.3	0.0539	4	9875.38	1014.45
0.5	0.0518	5	8291.97	1015.64
0.7	0.0509	5	7004.20	1015.59
0.9	0.0489	6	5949.16	1016

**Table 4:** Variation of profit for different *M* for Case 1

*M*	*N*	*n*	TP1	*Q*
35/365	0.0405	3	11,758.85	1006.68
40/365	0.0456	3	11,802.25	1008.47
45/365	0.0507	3	11,847.91	1010.55
50/365	0.0558	3	11,896.19	1013.02
55/365	0.0608	3	11,947.44	1015.14
60/365	0.0658	3	12,002.02	1019.14

**Table 5:** Results for different values of $a_2$ for Case 1

$a_2$	*N*	*n*	TP1	*Q*
30	0.0558	3	11,896.19	1013.02
50	0.0558	3	11,780.19	1013.02
100	0.0559	2	11,519.54	1011.01
150	0.0559	2	11,421.98	1011.01
200	0.0559	2	11,324.41	1011.01

## Conclusion

Here, we have proposed an EOQ model for deteriorating items considering two level credits where all customers are allowed to take the advantage of credit period from the retailer upto a limited period. The demand function is linked with customer’s credit period and the duration of publicity of such credit period in exponential nature. Here, retailer’s ordering cost per order has been considered as function of replenishment cycle number. Some examples have been provided to illustrate the model. Also, managerial insights have been carried out. The proposed model can be further extended in several ways like different demand structures, quantity discount, deterioration with lifetime, warranty cost and others.
